# Cutis Verticis Gyrata Across the Diagnostic Spectrum: Two Cases Highlighting Challenges in Clinical Classification

**DOI:** 10.1002/ccr3.73252

**Published:** 2026-08-04

**Authors:** Sanket Bishokarma, Shiwani Sharma Acharya, Goma Dhami

**Affiliations:** ^1^ Department of Dermatology, National Academy of Medical Sciences Bir Hospital Kathmandu Nepal

**Keywords:** cutis verticis gyrata, pachydermoperiostosis, primary essential cutis verticis gyrata, scalp folds, secondary cutis verticis gyrata

## Abstract

Cutis verticis gyrata (CVG) is an uncommon disorder characterized by cerebriform thickening of the scalp that may occur as a primary condition or secondary to a variety of systemic disorders. We report two patients with clinically distinct presentations of CVG illustrating the diagnostic challenges encountered during classification. A 40‐year‐old man presented with progressive scalp thickening and visual impairment, raising suspicion for primary non‐essential or secondary CVG; however, definitive classification was not possible because further ophthalmological and systemic investigations could not be completed after he was lost to follow‐up. A 28‐year‐old man presented with asymptomatic scalp folds accompanied by seborrhea and comedonal acne, initially suggesting pachydermoperiostosis. However, normal laboratory and endocrine investigations, including normal serum growth hormone levels, together with the absence of digital clubbing, periostosis, and radiographic abnormalities, favored primary essential CVG. These cases emphasize that CVG should be regarded as a clinical sign requiring systematic neurological, ophthalmological, endocrine, skeletal, and dermatological evaluation before definitive classification.

## Key Clinical Message

Cutis verticis gyrata is a clinical sign rather than a diagnosis. Careful evaluation for neurological, ophthalmological, endocrine, inflammatory, and genetic disorders is essential, as similar scalp morphology may arise from diverse underlying conditions requiring different diagnostic approaches.

## 1 Introduction

Cutis verticis gyrata (CVG) is an uncommon clinical finding characterized by cerebriform folds and furrows of the scalp. First described by Alibert in 1837 and later termed cutis verticis gyrata by Unna in 1907, it has a reported prevalence of approximately 1 in 100,000 males and is considerably less common in females [1].

CVG is traditionally classified into primary essential, primary non‐essential, and secondary forms. Primary essential CVG occurs in otherwise healthy individuals without associated abnormalities, whereas primary non‐essential CVG is associated with neurological, psychiatric, or ophthalmological disorders. Secondary CVG develops as a consequence of inflammatory dermatoses, endocrine disorders, connective tissue diseases, neoplasms, or genetic syndromes [2, 3].

Recent reports continue to emphasize that CVG should be regarded as a clinical sign rather than a distinct disease entity, requiring systematic evaluation for underlying neurological, ophthalmological, endocrine, inflammatory, neoplastic, and genetic disorders before definitive classification [4, 5]. Although individual cases representing different CVG subtypes have been reported, patients with incomplete or overlapping clinical features continue to pose significant diagnostic challenges. We report two such cases that illustrate the importance of cautious diagnostic interpretation and comprehensive evaluation before assigning an etiological classification.

## 2 Case 1

A 40‐year‐old man from Sindhupalchowk presented with progressive, asymptomatic thickening of the scalp for 2 years. He had additionally noticed prominent forehead folds on awakening during the preceding 2 weeks. The patient also reported gradually progressive diminution of vision over the previous 3 years.

There was no history of seizures, intellectual disability, psychiatric illness, scalp trauma, inflammatory scalp disease, endocrine disorders, or previous dermatological conditions involving the scalp. Family history was non‐contributory.

**FIGURE 1:**
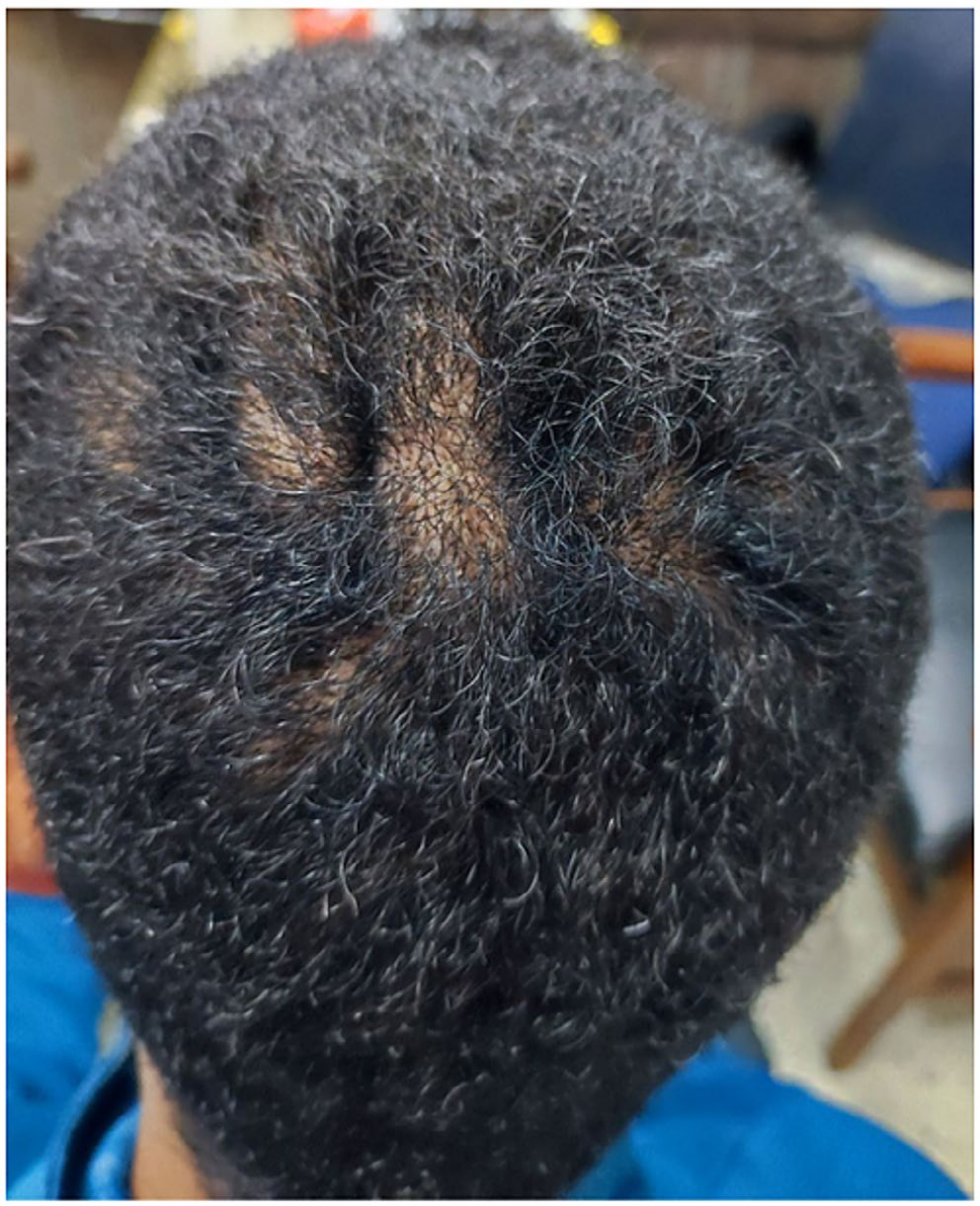
Case 1. Diffuse scalp thickening with multiple deep cerebriform folds and furrows involving the vertex and parietal scalp, consistent with cutis verticis gyrata.

General examination revealed stable vital signs and no systemic abnormalities. Cutaneous examination demonstrated diffuse scalp thickening with multiple deep convoluted folds and furrows arranged in a cerebriform pattern involving the vertex and parietal scalp (Figure 1). The folds were not reducible by pressure or traction. No digital clubbing, periostosis, palmoplantar keratoderma, or skeletal deformities were observed.

**FIGURE 2:**
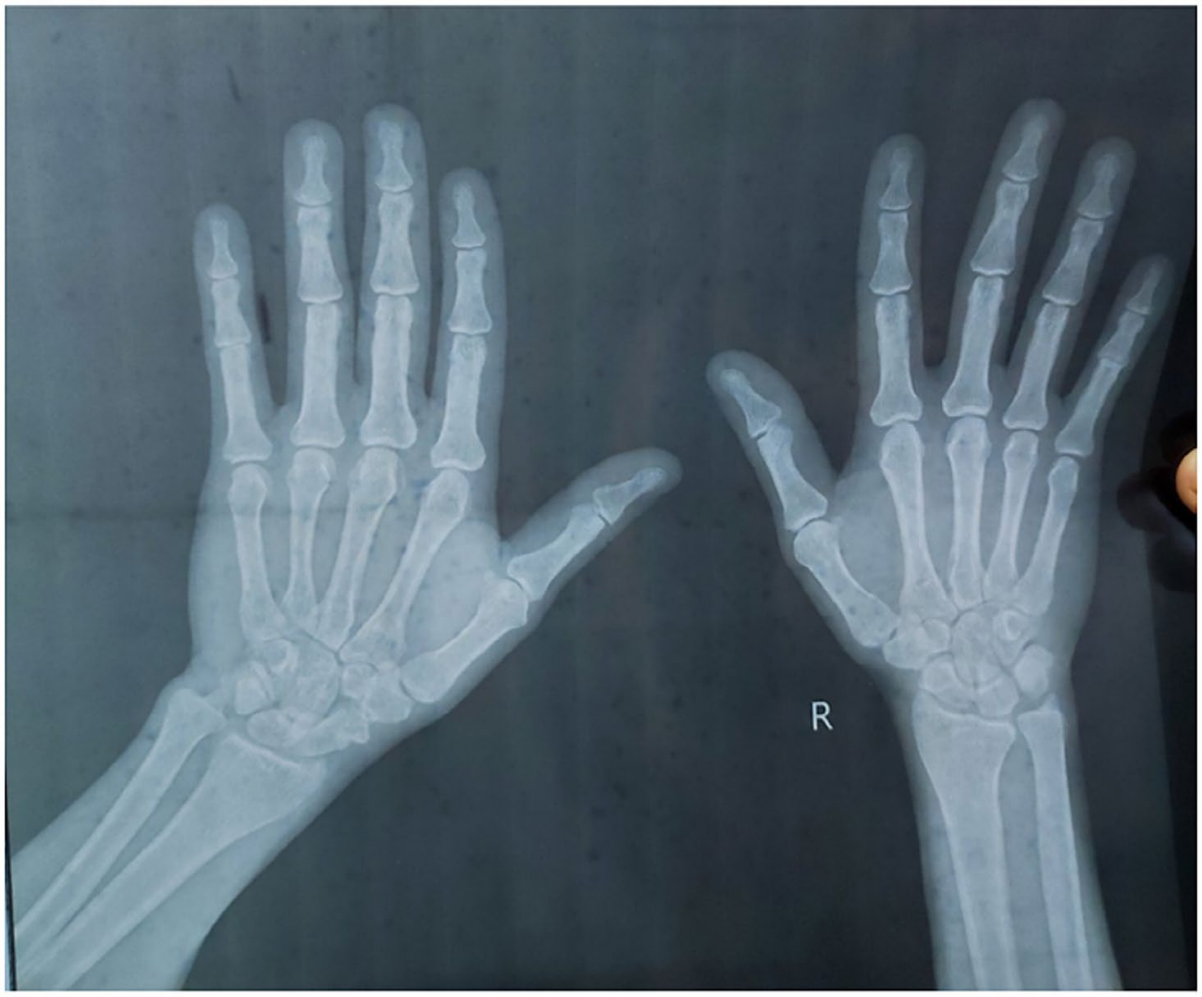
Case 1. Radiographs of the hands and forearms showing no evidence of periosteal reaction, cortical thickening, or other osseous abnormalities.

Anteroposterior radiographs of both hands and forearms were interpreted by a consultant radiologist and demonstrated no periosteal reaction, cortical thickening, or other osseous abnormalities (Figure 2). Routine laboratory investigations, including complete blood count, liver and renal function tests, blood glucose, thyroid function tests, and serum growth hormone evaluation, were requested as part of the diagnostic workup. However, the laboratory reports could not be retrieved because the patient was lost to follow‐up before completion of the evaluation.

The presence of associated visual impairment broadened the differential diagnosis beyond primary essential CVG and raised suspicion for primary non‐essential or secondary CVG. The patient was advised to undergo comprehensive ophthalmological and systemic evaluation; however, these investigations could not be completed because he was lost to follow‐up. Accordingly, the suspected classification remained provisional and could not be confirmed.

## 3 Case 2

A 28‐year‐old man presented with asymptomatic scalp folds first noticed 10 days earlier after hair trimming. He recalled undergoing haircuts previously without recognition of scalp abnormalities.

There was no history of arthralgia, bone pain, hyperhidrosis, flushing, weight loss, palmoplantar hyperkeratosis, or constitutional symptoms. Family history was negative for similar findings.

**FIGURE 3:**
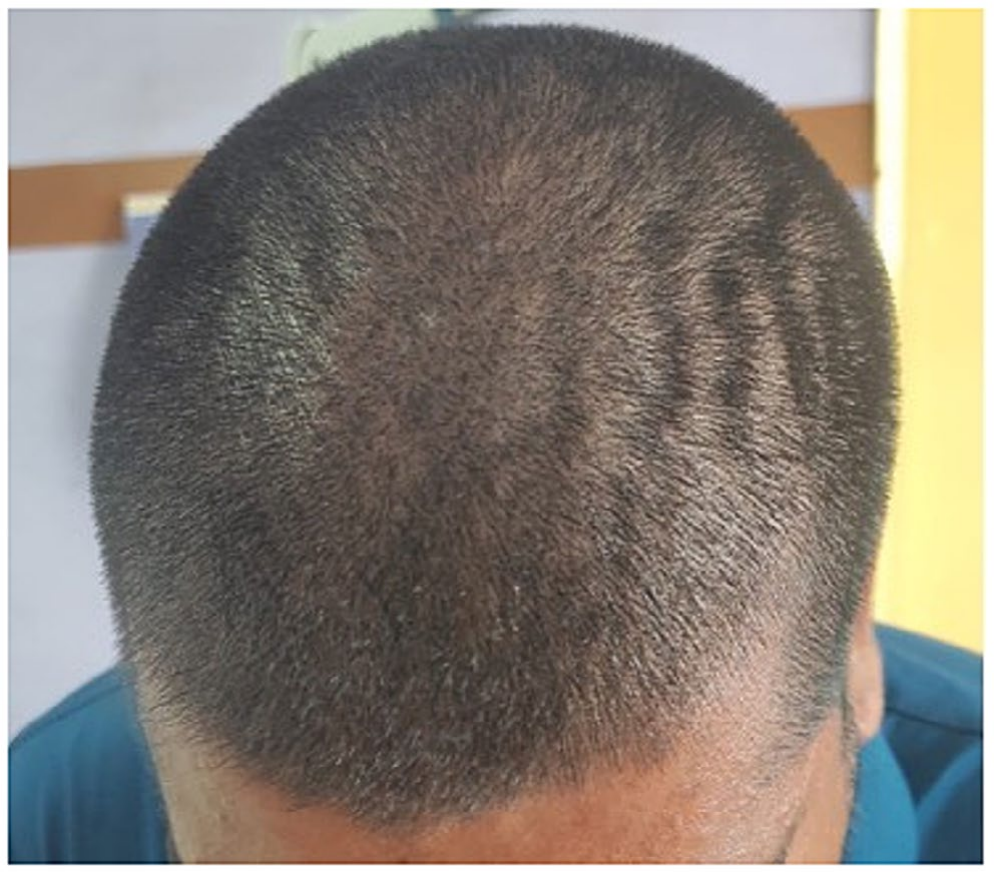
Case 2. Multiple thickened cerebriform folds and furrows involving the bilateral parietal scalp, more prominent on the left side.

Cutaneous examination revealed multiple thickened folds and furrows arranged in a cerebriform configuration over the bilateral parietal scalp, more prominent on the left side (Figure 3). The scalp was non‐tender and showed no evidence of inflammatory change. Facial examination demonstrated prominent seborrhea with multiple open and closed comedones and scattered inflammatory papules, consistent with acne vulgaris.

**FIGURE 4:**
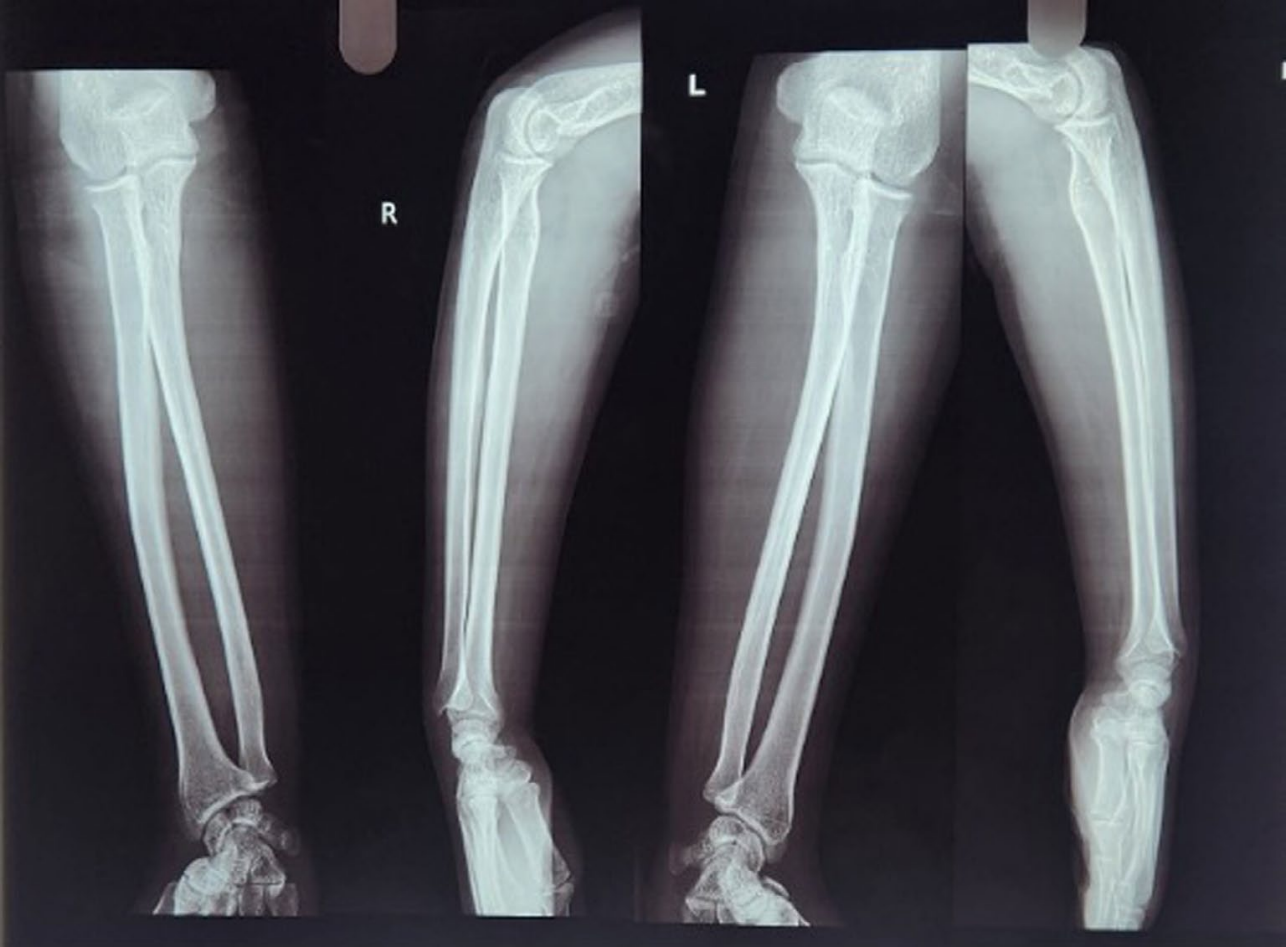
Case 2. Radiographs of the forearms and wrists showing absence of periosteal thickening, cortical irregularity, or other skeletal abnormalities.

No digital clubbing, periostosis, acropachy, or other skeletal abnormalities were detected. The palms and soles were normal. Anteroposterior radiographs of both wrists and forearms, reviewed in conjunction with the Department of Radiology by a consultant radiologist, demonstrated no periosteal reaction, cortical thickening, or other osseous abnormalities (Figure 4). Routine laboratory investigations, including complete blood count, liver and renal function tests, blood glucose, thyroid function tests, and serum growth hormone evaluation, were all within normal reference ranges, with no evidence of an underlying endocrine or systemic disorder.

Although the coexistence of seborrhea and acne vulgaris initially raised suspicion for an early or incomplete form of pachydermoperiostosis, the absence of digital clubbing, periostosis, hyperhidrosis, radiographic abnormalities, and laboratory evidence of an underlying endocrine disorder favored a diagnosis of primary essential cutis verticis gyrata. The application of established diagnostic criteria for pachydermoperiostosis and the rationale for its exclusion are discussed below.

## 4 Therapeutic Intervention

Both patients were counseled regarding the nature of the scalp findings and advised regarding further evaluation to exclude associated systemic disorders. No specific medical or surgical treatment was initiated.

## 5 Follow‐Up and Outcomes

Case 1 could not be reassessed because the patient was lost to follow‐up before completion of ophthalmological and systemic evaluation. At the most recent clinical review, Case 2 remained asymptomatic and showed no evidence of skeletal or systemic involvement. A summary of the clinical timeline for both patients is presented in Table 1.

**TABLE 1:** Timeline of clinical presentations of Case 1 and 2.

Time	Case 1	Case 2
3 years before presentation	Progressive visual impairment	—
2 years before presentation	Gradual scalp thickening	—
Prior years	—	Seborrhea and comedonal acne
10 days before presentation	—	Scalp folds noticed after haircut
Presentation	CVG diagnosed; radiographs normal	CVG diagnosed; radiographs normal
Follow‐up	Lost to follow‐up	Stable

## 6 Discussion

Our cases illustrate that the greatest diagnostic challenge in cutis verticis gyrata (CVG) is often not recognizing the characteristic scalp folds, but determining whether they represent primary disease or the manifestation of an underlying systemic disorder. Unlike many previously reported cases in which the underlying etiology was established, both patients presented with overlapping clinical features requiring cautious interpretation before classification. These cases therefore reinforce that CVG should be regarded as a descriptive morphological sign rather than a diagnosis in itself [2].

Primary essential CVG occurs in otherwise healthy individuals without associated neurological, psychiatric, ophthalmological, endocrine, or skeletal abnormalities, whereas primary non‐essential CVG is associated with neurological, psychiatric, or ophthalmological disorders [3, 6]. Secondary CVG develops in association with a wide range of inflammatory, endocrine, connective tissue, infiltrative, neoplastic, and genetic disorders, making systematic evaluation essential before assigning a final classification [2, 3].

The first patient presented with progressive scalp thickening accompanied by longstanding visual impairment, broadening the differential diagnosis beyond primary essential CVG and raising suspicion for primary non‐essential or secondary CVG. Ophthalmological abnormalities, including cataracts and retinal disorders, have previously been reported in association with primary non‐essential CVG [6]. However, because the patient was lost to follow‐up before completion of the planned ophthalmological and systemic evaluation, the suspected classification remained provisional and definitive categorization was not possible. This diagnostic uncertainty should be considered when interpreting the findings of the first case and further emphasizes the importance of complete multidisciplinary assessment before classification. The transient prominence of the forehead folds reported on awakening could not be further evaluated because the patient was lost to follow‐up, and its clinical significance therefore remains uncertain.

The second patient highlights the challenge of distinguishing primary essential CVG from early or incomplete pachydermoperiostosis (PDP). PDP is characterized by pachydermia, digital clubbing, periostosis, seborrhea, hyperhidrosis, acne, and occasionally CVG [7]. Although our patient exhibited seborrhea and comedonal acne, he did not fulfill established diagnostic criteria for PDP. According to the Touraine‐Solente‐Golé classification, the absence of digital clubbing and periostosis excluded the complete and incomplete forms of PDP, while normal radiographs demonstrated no skeletal involvement. Similarly, the patient did not fulfill the clinical diagnostic criteria proposed by Borochowitz et al. Furthermore, routine laboratory investigations, including complete blood count, liver and renal function tests, blood glucose, thyroid function tests, and serum growth hormone evaluation, were within normal reference ranges, making endocrine disorders such as acromegaly less likely. In addition to PDP, other differential diagnoses considered included cerebriform intradermal nevus, inflammatory scalp disorders (particularly psoriasis and chronic eczema), myxedema, amyloidosis, and infiltrative or neoplastic disorders that may produce secondary CVG [5, 8, 9]. The absence of characteristic clinical features, localized scalp lesions, skeletal abnormalities, or other systemic manifestations made these diagnoses less likely in our patients, although complete exclusion of all secondary causes was not possible in Case 1 because the diagnostic evaluation remained incomplete. Continued clinical follow‐up would nevertheless be advisable to identify any evolving manifestations that might warrant reclassification [10, 11].

Secondary CVG has been reported in association with acromegaly, myxedema, amyloidosis, cerebriform intradermal nevus, leukemia, lymphoma, inflammatory dermatoses, and connective tissue disorders [8, 12]. Consequently, the appearance of cerebriform scalp folds should prompt systematic neurological, ophthalmological, endocrine, skeletal, and dermatological evaluation rather than immediate classification as primary disease.

An important lesson illustrated by these cases is that CVG should be regarded as a clinical sign rather than a diagnosis. Similar to clubbing or xanthelasma, its recognition should prompt systematic evaluation for associated neurological, ophthalmological, endocrine, inflammatory, neoplastic, and genetic disorders before assigning a definitive classification. Recent reports continue to emphasize the importance of a multidisciplinary approach and careful assessment for underlying comorbidities [4, 5]. The contrasting presentations observed in our patients further illustrate the broad diagnostic spectrum encompassed by this uncommon condition.

Comparison with previously published reports (Table 2) demonstrates that similar cerebriform scalp folds may occur in markedly different clinical contexts, reinforcing the importance of systematic investigation before assigning a definitive classification.

**TABLE 2:** Comparison of the present cases with selected published reports of cutis verticis gyrata.

Study	Clinical presentations	Key investigations	Final diagnosis	Principal clinical message
Larsen and Birchall (2007) [3]	Three patients representing different clinical forms of CVG	Clinical, neurological, and systemic evaluation	Primary essential, primary non‐essential, and secondary CVG	CVG should be classified only after careful evaluation for associated disorders.
Chamli et al. (2022) [4]	Three patients with different underlying etiologies of CVG	Clinical examination with targeted laboratory, imaging, and histopathological investigations	Three distinct causes of CVG	Identification of the underlying etiology guides diagnosis and management.
Devkota and Bhatta (2025) [5]	Incidentally detected asymptomatic CVG	Clinical assessment and neuroimaging	Primary essential CVG	Primary essential CVG is a diagnosis of exclusion after appropriate systemic evaluation.
Present Case 1	CVG with progressive visual impairment	Clinical examination; skeletal radiographs normal; planned ophthalmological and systemic evaluation could not be completed because the patient was lost to follow‐up	Provisional primary non‐essential or secondary CVG	Visual symptoms should prompt comprehensive ophthalmological and systemic evaluation. Incomplete investigations preclude definitive classification.
Present Case 2	CVG with seborrhea and comedonal acne, raising suspicion for early/incomplete pachydermoperiostosis	Clinical examination; skeletal radiographs; complete blood count; liver and renal function tests; blood glucose; thyroid function tests; serum growth hormone evaluation	Primary essential CVG	Early or incomplete pachydermoperiostosis should be considered in patients with seborrhea or acne. Systematic clinical, radiographic, and laboratory evaluation is essential before excluding secondary causes.

Management depends on the underlying etiology. In primary essential CVG, reassurance and maintenance of scalp hygiene are generally sufficient, while surgical correction may be considered for cosmetic concerns or difficulty maintaining scalp hygiene [2]. In secondary CVG, identification and treatment of the underlying disorder remain the cornerstone of management.

## 7 Limitations

A major limitation of this report is the inability to complete the planned ophthalmological and systemic evaluation in Case 1 because the patient was lost to follow‐up. Consequently, the suspected classification remained provisional, and definitive classification as primary non‐essential or secondary cutis verticis gyrata could not be established. This diagnostic uncertainty should be considered when interpreting the findings of the first case. Additionally, the absence of longitudinal follow‐up limited assessment of disease progression and the potential emergence of systemic features that might have further refined the diagnostic classification.

## 8 Conclusion

These two cases highlight that the principal clinical challenge in cutis verticis gyrata (CVG) lies not in recognizing the characteristic cerebriform scalp folds, but in accurately determining their underlying etiology. Similar clinical morphology may represent primary essential disease, primary non‐essential disease, or secondary CVG associated with systemic disorders. Our cases illustrate how incomplete or overlapping clinical features can complicate diagnostic classification and reinforce that CVG should be regarded as a clinical sign rather than a diagnosis. A systematic evaluation, including neurological, ophthalmological, endocrine, skeletal, and other relevant assessments, is essential before assigning a definitive classification. Careful multidisciplinary assessment remains the cornerstone of appropriate diagnosis and management.

## 9 Patient Perspective

The patient (Case 2) stated that he was initially anxious after noticing the scalp folds but felt reassured once the benign nature of the condition and the need for systematic evaluation were explained. He expressed satisfaction with the proposed management plan and agreed to clinical follow‐up. Another patient (Case 1): Not available because the patient was lost to follow‐up.

## Author Contributions

**Shiwani Sharma Acharya:** writing – original draft, writing – review and editing. **Goma Dhami:** conceptualization, writing – review and editing, supervision. **Sanket Bishokarma:** writing – original draft, writing – review and editing, data curation, conceptualization.

## Funding

The authors have nothing to report.

## Consent

Written informed consent was obtained from both patients for publication of clinical information and images.

## Conflicts of Interest

The authors declare no conflicts of interest.

## Data Availability

Data supporting the findings of this report are available from the corresponding author upon reasonable request.
